# Bioactive small molecules produced by the human gut microbiome modulate *Vibrio cholerae* sessile and planktonic lifestyles

**DOI:** 10.1080/19490976.2021.1918993

**Published:** 2021-05-19

**Authors:** Heidi Pauer, Felipe Lopes Teixeira, Avery V. Robinson, Thiago E. Parente, Marília A. F. De Melo, Leandro A. Lobo, Regina M. C. P. Domingues, Emma Allen-Vercoe, Rosana B. R. Ferreira, Luis Caetano M. Antunes

**Affiliations:** a Instituto Nacional de Ciência e Tecnologia de Inovação Em Doenças De Populações Negligenciadas, Centro De Desenvolvimento Tecnológico em Saúde, Fundação Oswaldo Cruz, Rio de Janeiro, Brazil; b Departamento de Tecnologia Farmacêutica, Universidade Federal Fluminense, Niterói, Brazil; c Department of Molecular and Cellular Biology, University of Guelph, Guelph, Canada; d Laboratório de Genômica Funcional e Bioinformática, Instituto Oswaldo Cruz, Fundação Oswaldo Cruz, Rio de Janeiro, Brazil; e Departamento de Microbiologia Médica, Instituto de Microbiologia Paulo de Góes, Universidade Federal do Rio De Janeiro, Rio de Janeiro, Brazil; f Escola Nacional de Saúde Pública Sergio Arouca, Fundação Oswaldo Cruz, Rio de Janeiro, Brazil; g Laboratório de Pesquisa Em Infecção Hospitalar, Instituto Oswaldo Cruz, Fundação Oswaldo Cruz, Rio de Janeiro, Brazil

**Keywords:** Gut metabolome, Antivirulence, Bioactive small molecules, *Vibrio cholerae*, Microbial signaling, Motility

## Abstract

Humans live in symbiosis with a diverse community of microorganisms, which has evolved to carry out many specific tasks that benefit the host, including protection against invading pathogens. Within the chemical diversity of the gastrointestinal tract, small molecules likely constitute chemical cues for the communication between the microbiota and pathogens. Therefore, we sought to investigate if molecules produced by the human gut microbiota show biological activity against the human pathogen *Vibrio cholerae*. To probe the effects of the gut metabolome on *V. cholerae*, we investigated its response to small-molecule extracts from human feces, from a complex bacterial community cultivated *in vitro*, and from culture supernatants of *Enterocloster citroniae, Bacteroides thetaiotaomicron*, and *Bacteroides vulgatus*. Using RNA sequencing, we determined the impact of the human gut metabolome on *V. cholerae* global gene expression. Among the genes downregulated in the presence of the fecal extract, the most overrepresented functional category was cell motility, which accounted for 39% of repressed genes. Repression of *V. cholerae* motility by the fecal extract was confirmed phenotypically, and *E. citroniae* extracts reproduced this phenotype. A complex *in vitro* microbial community led to increased motility, as did extracts from *B. vulgatus*, a species present in this community. Accordingly, mucin penetration was also repressed by fecal and *E. citroniae* extracts, suggesting that the phenotypes observed may have implications for host colonization. Together with previous studies, this work shows that small molecules from the gut metabolome may have a widespread, significant impact on microbe–microbe interactions established in the gut environment.

## Introduction

The importance of the gut microbiota for human health is well known, but the molecular mechanisms involved are not fully elucidated. The microbiota contributes to many of the biochemical pathways found in the human gut, aiding in the utilization of proteins and indigestible polysaccharides as carbon and energy sources by breaking them into essential amino acids and short chain fatty acids.^1^ As the majority of microbiota members are metabolically active, they are constantly producing and secreting small molecules.^2^ These molecules are part of the gut metabolome, the collection of all small molecules (<3000 Da) present in the gastrointestinal tract, and form a chemical framework that can exert many functions in this environment.^3–5^ Interactions between host and gut microbiota contribute significantly to the production of small molecules that can be excreted into the feces or reach various organs and tissues through systemic blood circulation.^6,7^ The effects of such chemical interactions are still mostly unknown, although some compounds can affect host cell differentiation and metabolism.^8,9^

Over the past few years, studies have demonstrated the interference of small molecules in the survival and behavior of pathogenic species, thus providing a new tool for understanding their relationships with the human host.^10^ Our group has recently shown that aromatic compounds from the human gut metabolome have a significant impact on the virulence of *Salmonella enterica* serovar Typhimurium.^11,12^ Other groups have made similar observations with other enteric pathogens, such as *Clostridioides difficile* and enteropathogenic *Escherichia coli*.^13–15^ However, many other human pathogens are likely to have evolved mechanisms to respond to chemical cues from the resident microbiome, although there have been very few studies investigating the effect of the gut metabolome on other pathogens.

*Vibrio cholerae* is the causative agent of the intestinal disease, cholera. The main source of infection is contaminated water and food, and thus cholera is an important disease in many developing countries within Africa, Asia and South America that suffer from poor water supplies and sanitation.^16^ *V. cholerae* is a noninvasive bacterium that affects the small intestine through epithelial cell adhesion and production of an enterotoxin, cholera toxin (CT).^17^ ToxR and other regulatory proteins regulate CT expression, and several virulence genes within the ToxR regulon are involved in cholera disease. In addition to the essential role of CT in cholera, the toxin-coregulated pilus (TCP) is pivotal for the colonization of the intestinal epithelium and helps in microcolony formation on the epithelial surface.^16,18^

Humans, their microbiota, and intestinal pathogens have co-evolved for hundreds of thousands of years. Thus, it is probable that chemical signaling between the intestinal microbiota and pathogens represent not an exception, but rather the rule governing the interactions between these beneficial microbial communities and invaders. The role of the gut microbiota in protection against invading pathogens has been studied for decades, and several studies suggest that small molecules may be involved in this phenomenon.^11,12,19,20^ In this study, we set out to determine whether the human gut metabolome has biological activity that mitigates *V. cholerae* virulence by examining pathogen gene expression and behavior in response to small molecules produced by members of the gut microbiota. Our results showed that the gut metabolome exerts specific effects on pathogen gene expression, and that these effects translate into phenotypic changes. How each member of the microbiota and the molecules they produce integrate to control the dynamics of microbiota–pathogen interactions in this complex environment will be an exciting area of research for years to come.

## Materials and methods

A scheme displaying the methods employed in this work is presented in Figure S1.

### Ethics statement

This study was carried out in accordance with the recommendations and approval of the Research Ethics Committee of the *Escola Nacional de Saúde Pública Sergio Arouca, Fundação Oswaldo Cruz* (protocol number 69742817.6.0000.5240).

### Bacterial strains and growth conditions

The *V. cholerae* strain used in this work was VC833, a multidrug-resistant *V. cholerae* O1 strain isolated from human feces during a cholera outbreak in Nigeria in 2010.^21^ The strain was obtained from the Bacteria Culture Collection of Environment and Health at the *Fundação Oswaldo Cruz*. VC833 is resistant to streptomycin, trimethoprim, trimethoprim/sulfamethoxazole, cefotin, cefuroxime, ampicillin, nalidixic acid, ciprofloxacin, sulfonamide, sulfamethoxazole, and chloramphenicol.^21^ *V. cholerae* was routinely grown in Brain Heart Infusion broth (BHI; Sigma-Aldrich) supplemented with hemin (5 mg/mL; Sigma-Aldrich) at 37°C with shaking (225 rpm).

*Bacteroides thetaiotaomicron* ATCC29741 and *Bacteroides vulgatus* ATCC8482 were obtained from the American Type Culture Collection. *Enterocloster* (previously *Clostridium*) *citroniae* FM-V5-E was isolated from a stool sample from a healthy 38-year old female.^11^ *Bacteroides* and *E. citroniae* strains were cultured in an anaerobic chamber (80% N_2_, 10% H_2_, 10% CO_2_) in BHI (Sigma-Aldrich) supplemented with hemin (5 mg/mL; Sigma-Aldrich) at 37°C.

### Chemostat

A 124-strain, 69-species community was cultured *in vitro* within a continuously fed bioreactor and was compiled from a collection of previously-isolated strains originating from a stool sample of a 41-year-old female.^22^ Strains were routinely grown on Fastidious Anaerobe Agar (Neogen) supplemented with 5% v/v defibrinated sheep’s blood (Hemostat Laboratories) (FAA) within an AS-580 Anaerobe Chamber (Anaerobe Systems) in an atmosphere of N_2_/CO_2_/H_2_ 90:5:5. Before inclusion into the community, strains were streaked to purity on FAA, followed by V3-V4 variable region 16S rRNA gene Sanger sequencing for species verification (Table S2). Individual strains were then frozen at −80°C for storage in skim milk freezing media supplemented with dimethyl sulfoxide (Thermo Scientific). Community stocks for chemostat seeding were created by combining equivalent volumes of each strain grown in a lawn before freezing at −80°C for storage in skim milk freezing media without dimethyl sulfoxide.

The chemostat, a 500-mL Multifors bioreactor with a working volume of 400 mL (Infors), had conditions set to mimic the human colonic lumen: 37°C, pH 7, gentle agitation, anaerobic under nitrogen gas bubbling, and supplied continually with a medium designed to emulate the effluent from the small intestine (Table S3).^23^ The 5 mL community stock was thawed from −80°C to room temperature within the Anaerobe Chamber before adding to 400 mL of degassed medium within the chemostat. The community was allowed 24 h in batch culture within the chemostat before fresh medium was added or waste removed, to allow an increase in biomass. The bioreactor vessel was operated and maintained as previously described.^23^

The vessel was maintained for 21 days before the entire contents were harvested. Aliquots of 35 mL of this material were centrifuged at 15,000 g and 4°C for 30 min prior to 0.22 µm filter-sterilization. After that, 330 mL of the community filtrate was aliquoted and snap-frozen in ethanol and dry ice followed by overnight lyophilization in a FreeZone 4.5 Liter Benchtop Freeze Dry System (Labconco). The lyophilized community filtrate was reconstituted in water before further investigation.

### Human fecal samples

Fecal samples were collected from a 35-year-old female with no history of significant gastrointestinal problems and who did not undergo antibiotic treatment for at least six months before sample collection. Fresh feces were collected using a sterile container, refrigerated and brought to the laboratory within 24 h, where they were immediately used or frozen at −20°C until used.

### Small-molecule extraction

To extract small molecules from human feces, thereby producing the fecal extracts used in this work, stool samples were first weighed and transferred to a sterile glass vessel. Then, ethyl acetate (Sigma-Aldrich) was added in a 1:1 (w/v) ratio. The mixture was vortexed for 5 min and then incubated with shaking (100 rpm) for 18 h. After the incubation, the liquid phase was collected in a glass flask and the extract was evaporated in a rotary evaporator (Heidolph) and then frozen at −20°C until used. As a control, ethyl acetate was also dried in a rotary evaporator and the sediment was used in experiments.

In order to obtain small-molecule extracts from *E. citroniae, B. thetaiotaomicron* and *B. vulgatus* cultures, bacterial strains were inoculated in 250 mL of BHI and incubated in anaerobiosis at 37°C for 18 h. After incubation, 250 mL of ethyl acetate was added to the cultures and the vials were shaken vigorously and then allowed to stand for 10 min. The vials were then vigorously mixed again and left for another 10 min at rest to allow phases to separate. The organic phase was collected and dried in a rotary evaporator. As a control, BHI medium without bacterial growth was also extracted, as described. Dried extracts were maintained at −20°C until used. Chemostat effluent was processed in the same manner. First, freeze-dried effluent was reconstituted in the original volume of water to bring metabolites up to their original concentration. Then, one volume of ethyl acetate was added and the molecules were extracted as described above for bacterial cultures. As a control, chemostat media without bacterial growth was also extracted, as described.

For experiments using small-molecule extracts, the dried material was suspended directly in BHI. The suspension was filtered (0.45-μm pore size) to remove larger particles that did not solubilize and the pH was adjusted to 7.2, followed by filtration again using 0.2-μm filters to sterilize extracts. Fecal extracts were used in experiments at a 1x relative concentration, to approximate the metabolite concentrations found in feces. Bacterial culture extracts were used at a 2x relative concentration in all experiments.

### Bacterial growth curves

An overnight culture of *V. cholerae* was diluted 1:200 in BHI broth, with or without the addition of extracts from human feces, *B. thetaiotaomicron, E. citroniae, B. vulgatus* or chemostat cultures, and added to a 96-well microplate. The growth curve was performed using the Infinite® F50 spectrophotometer (TECAN) with readings every 30 min for 12 h at an optical density of 620 nm (OD_620_).

### RNA sequencing and data analysis

*V. cholerae* was grown in BHI broth with and without the fecal extract. After approximately 4 h of growth (mid-log phase), bacterial RNA was stabilized by the addition of 2 volumes of RNAprotect bacterial reagent (Qiagen) to bacterial cultures, which were thoroughly mixed and incubated at room temperature for approximately 5 min. Cells were pelleted by centrifugation, and RNA was isolated using the RNeasy Mini Kit (Qiagen), according to the manufacturer’s recommendations. RNA was quantified using a NanoVue spectrophotometer (GE HealthCare). RNA was treated with Turbo DNase (2 U/μL) according to the manufacturer’s manual (Life Technologies) to eliminate residual DNA, followed by purification using the RNeasy Mini kit (Qiagen) according to the manufacturer’s recommendations. rRNA depletion was performed with the Ribo-Zero rRNA Removal Kit (Illumina), according to the manufacturer´s recommendations (Document #15,066,012 v02). Library construction was performed with the TruSeq Stranded mRNA Sample Preparation Kit, according to the manufacturer´s recommendations (catalog #RS-122-9004DOC). The final libraries obtained were assessed for quality using a DNA 1000 chip on an Agilent Technologies 2100 Bioanalyzer (Agilent) and quantified using Qubit 2.0 kit High Sensitivity (ThermoFisher). Sequencing was performed on an Illumina HiSeq 2500 (Illumina) at the *Plataforma de Sequenciamento de Ácidos Nucleicos de Nova Geração – RPT01J* of the *Fundação Oswaldo Cruz* (Rio de Janeiro, Brazil).

Data processing and analyses were performed at the *Plataforma de Bioinformática – RPT04A* of the *Fundação Oswaldo Cruz*, as previously described.^24^ In summary, raw sequencing read files (bcl files) were converted to fastq files with bcl2fastq software version 2.17 (Illumina). Technical and low-quality sequences were removed using Trimmomatic (v 2.2.0) with the following parameters: ILLUMINACLIP:TruSeq3-PE-2.fa:2:30:10 LEADING:3 TRAILING:3 SLIDINGWINDOW:4:20 MINLEN:40.^25^ Read quality was assessed using FastQC (Babraham Bioinformatics), and filtered reads were mapped with Bowtie2 (–no-mixed – no-discordant) to the two chromosomes of *V. cholerae* O1 biovar El Tor str. N16961 (GenBank accession no. NC_002505 and NC_002506) as references.^26^ SAMtools was used to extract and sort mapped reads (-G 77, -G 141), and the Cufflinks suite was used to compare global gene expression between conditions.^27–29^ Genes that showed a differential expression of 2-fold with *p*< .05 between conditions (absence or presence of fecal extract) were considered significantly regulated. Genes were functionally annotated and had their functional abundance profiles created with EggNOG (v. 5.0.0) (http://eggnog5.embl.de).^30^ Protein-protein association networks of significantly altered genes (≥10-fold change) were analyzed using STRING v11.0 (https://string-db.org/).^31^

### Motility assays

Motility assays were carried out in BHI medium containing 0.3% agar with or without fecal, *E. citroniae, B. thetaiotaomicron, B. vulgatus* or chemostat extracts. Culture medium was poured into Petri dishes and allowed to solidify overnight. Plates were then inoculated with a single colony using a sterile toothpick and incubated at 37°C. The diameter of the motility halo was measured after 18 h.

### Mucin penetration assays

Mucin penetration assays were performed in 1% mucin columns (Mucin from porcine stomach, type II, Sigma) in 1 mL syringes. *V. cholerae* was grown with or without the addition of extracts from human feces, *E. citroniae* or *B. thetaiotaomicron* cultures for approximately 4 h, until the mid-log growth phase was reached. Cells were then pelleted, washed, and reconstituted in phosphate-buffered saline (pH 7.4), and the suspension standardized to an OD_600_ of 0.3. A 0.1 mL aliquot of the cell suspension was added to the top of 1% mucin columns. Columns were kept at 37°C in vertical position under static conditions. After 30 min of incubation, 500 µL fractions were collected from the bottom of the columns, serially diluted, and plated onto BHI agar to measure CFU after incubation at 37°C for 16 h.^32^

### Biofilm formation assays

Quantification of total growth and biofilm formation was performed as described previously, with some modifications. Briefly, *V. cholerae* strains were grown overnight in LB broth (1% NaCl) at 37°C with shaking (250 rpm). Following overnight growth, the culture was diluted 1:200 in LB broth with and without the addition of extracts from human feces, *E. citroniae* or *B. thetaiotaomicron* cultures and grown as static cultures in a 96-well polystyrene plate (Greiner) at 37°C. After incubation for approximately 48 h, planktonic cell suspensions were collected, leaving only surface-attached cells in each well. The planktonic cell density was determined by measuring the OD_600_. To quantify biofilm formation, 200 μL of PBS were added to the surface-attached cells remaining in the microplate, and the cells were dispersed by vigorous pipetting and scraping. The amount of biofilm formed was determined by measuring the OD_600_ of the resulting cell suspension using an Infinite M200 PRO microplate reader (TECAN). The reported total growth was calculated from the sum of the OD_600_ values measured for planktonic and biofilm cell suspensions.

## Results

### The human gut metabolome moderately inhibits V. cholerae growth

**Figure 1.**
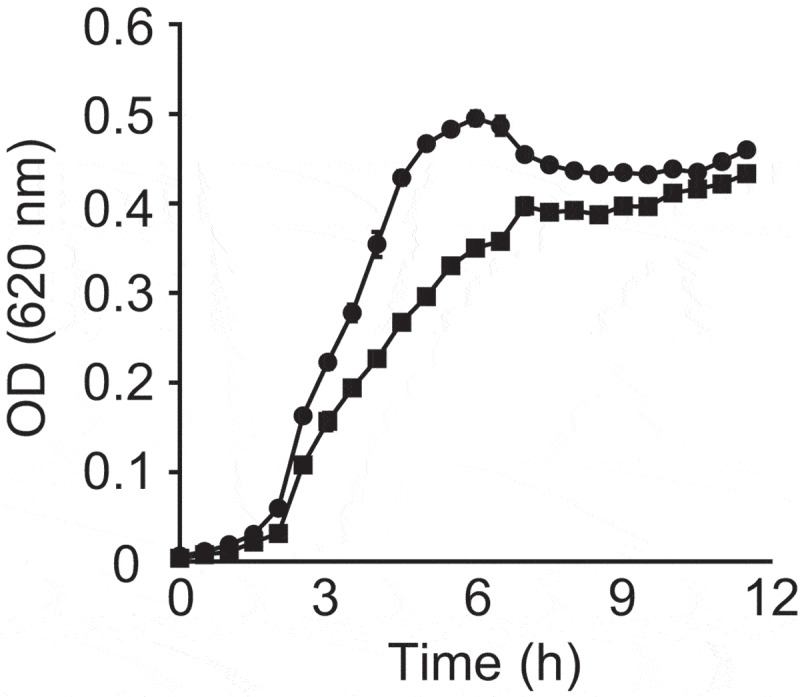
Impact of a fecal extract on *V. cholerae* growth *in vitro.*

In order to test if small molecules extracted from fresh feces of a healthy donor have any effect on *V. cholerae*, we compared bacterial growth with or without the fecal extract. As can be observed in Figure 1, *V. cholerae* growth was slightly delayed in the presence of the fecal extract. In regular culture medium, *V. cholerae* reached stationary phase after approximately 5.5 hours of growth. Growth in the fecal extract led to a delay of approximately 1.5 hours, with *V. cholerae* reaching stationary phase after approximately 7 hours. Although the optical density (OD) reached by the cultures in stationary phase was comparable, a significant difference was observed during exponential growth, with the largest difference being 36.6% at 5 hours of growth (doubling time of 52.72 ± 1.70 minutes for control cultures *vs*. 61.64 ± 7.03 minutes for cultures containing the extract). This reduction was not caused by either pH differences or solvent impurities, since the pH of the culture medium containing the fecal extract was adjusted to match that of the control medium, and the control cultures contained an ethyl acetate evaporate without fecal material. This result suggests that molecules of low polarity present in the fecal metabolome show moderate biological activity against *V. cholerae* growth. However, both cultures in the presence and absence of the fecal extract reached similar OD values in the stationary phase.

Cultures were grown in BHI broth with or without fecal extracts at 37 °C and growth was measured by absorbance (OD_620_) for 12 h in a microplate reader. Results represent the average of three independent cultures (n = 3), and bars show the standard errors of means. Circles indicate growth in the absence of the fecal extract, whereas squares indicate cultures with the extract.

### Small molecules from human fecal extracts modulate V. cholerae gene expression

**Figure 2.**
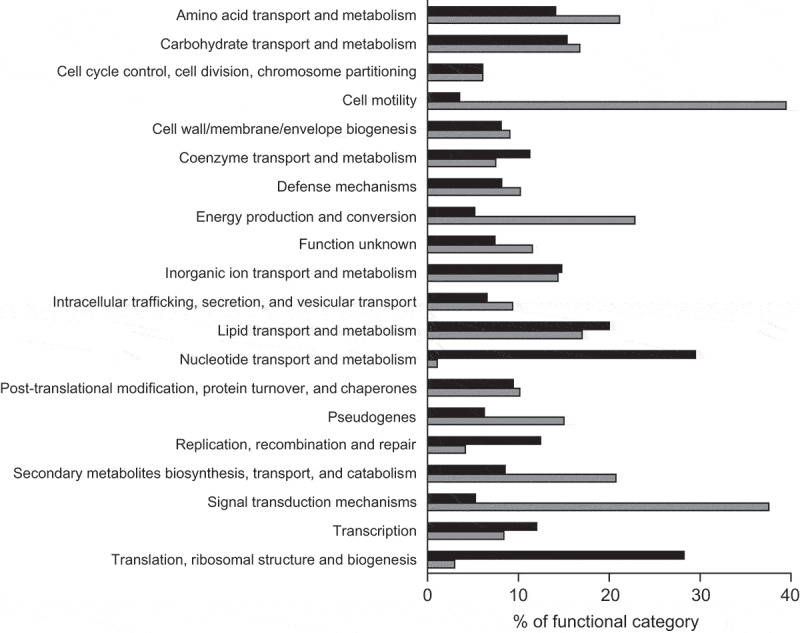
Functional analysis of *V. cholerae* genes differentially expressed during growth in the presence of a human fecal extract

**Figure 3.**
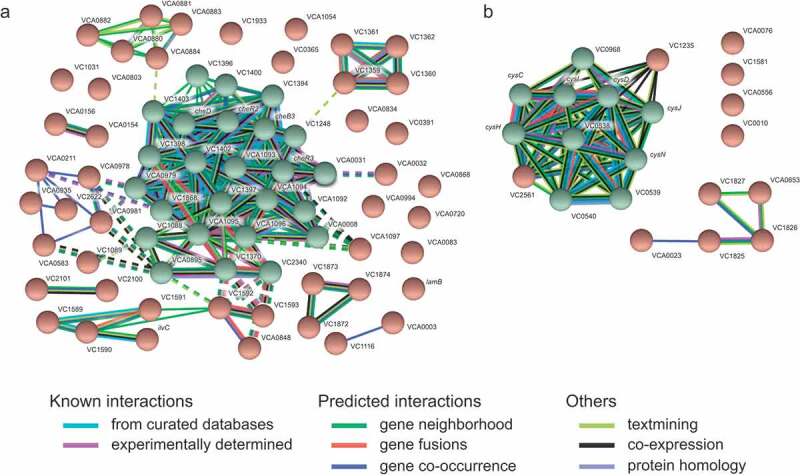
STRING analysis of predicted interactions between proteins encoded by genes differentially regulated by the fecal extract

To further investigate the effect of the human gut metabolome on *V. cholerae*, we compared the transcriptome of cells during mid-log growth in the absence or presence of a human fecal extract. RNAseq results revealed 912 differentially expressed genes, representing 24.4% of all genes in the genome. The results of the transcriptome analysis can be seen in Table S1, which shows expression levels of all genes. Specifically, 410 genes were upregulated, whereas 502 genes were downregulated during growth in the presence of the fecal extract. We grouped these genes into functional categories based on proposed functions using EggNOG. As shown in Figure 2, among genes upregulated by the fecal extract, the two most altered functional categories were nucleotide transport and metabolism (29%), and translation, ribosomal structure, and biogenesis (28%). Among downregulated genes, cell motility (39%), and signal transduction mechanisms (37%) were the two most regulated functional categories (Figure 2). Also, to identify gene categories whose genes were most affected by the fecal extract, an interaction network of proteins coded by genes whose expression was altered at least tenfold was constructed. We observed that among the 69 genes downregulated at least tenfold, 25 are involved in *V. cholerae* chemotaxis (Figure 3a). A network of proteins coded by genes upregulated at least tenfold by the fecal extract was also constructed and revealed that several of them are related to sulfur metabolism (Figure 3b).

Following RNAseq, genes with altered expression (twofold or more) were allocated in groups based on their proposed functions according to EggNOG (5.0.0). Black bars represent % of genes upregulated whereas gray bars indicate % of genes downregulated during growth in medium containing a fecal extract.

A. Network of genes downregulated at least tenfold during growth in the presence of the fecal extract. Green circles indicate a cluster of genes related to chemotaxis and motility in *V. cholerae*, with red circles representing genes unrelated to these functions. B. Network of genes upregulated at least tenfold during growth in the presence of the fecal extract. Green circles indicate genes related to sulfur metabolism, whereas red circles represent genes in other categories.

**Figure 4.**
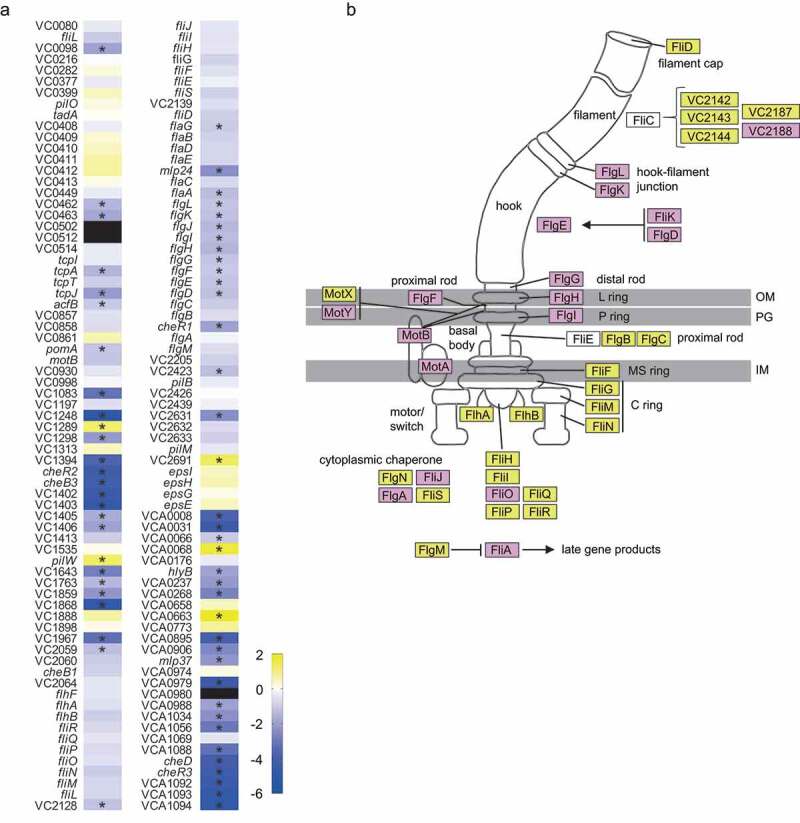
Effect of the fecal extract on the expression of *V. cholerae* motility-associated genes

Given that there was a clear overrepresentation of motility and chemotaxis predicted functions within the group of genes regulated by the fecal extract, we next assessed the magnitude of the observed effect on motility and chemotaxis genes. Figure 4a shows a heatmap of 142 genes associated with motility, as predicted by EggNOG, of which 63 genes were significantly (≥2-fold, *p*< .05) affected. Among them are genes related to chemotaxis, mainly cluster I (*cheR2, cheB3*, VC1402, VC1403) and cluster III (*cheD, cheR3*, VCA1092, VCA1093, VCA1094) genes. In addition to genes related to chemotaxis, genes related to the flagellum were also modulated, including *flaA*, required for filament synthesis. Figure 4b depicts a flagellar model from KEGG where the effect (or lack thereof) of the fecal extract on the expression of *V. cholerae* genes is indicated, once again showing a marked repressive effect of the human fecal metabolome on flagellar expression.

A. Heat map of fold changes (Fecal extract/Control; log2) of all genes annotated as being involved with *V. cholerae* motility in the absence and presence of the fecal extract. Blue represents genes whose expression was repressed by the extract, yellow indicates genes whose expression was activated by the extract, and white are genes whose expression was unchanged. Black indicates genes that were not expressed in either condition. Asterisks indicate statistical significance (*p*< .05). B. Flagellar assembly scheme depicting proteins encoded by genes whose expression was affected by the fecal extract. Purple indicates genes whose expression levels were repressed by the fecal extract by 2-fold or more. Yellow indicates genes repressed between 1.3 and 2-fold, whereas genes whose expression was not significantly altered are depicted in white.

### Bioactive molecules from the human gut inhibit V. cholerae motility and mucin penetration

**Figure 5.**
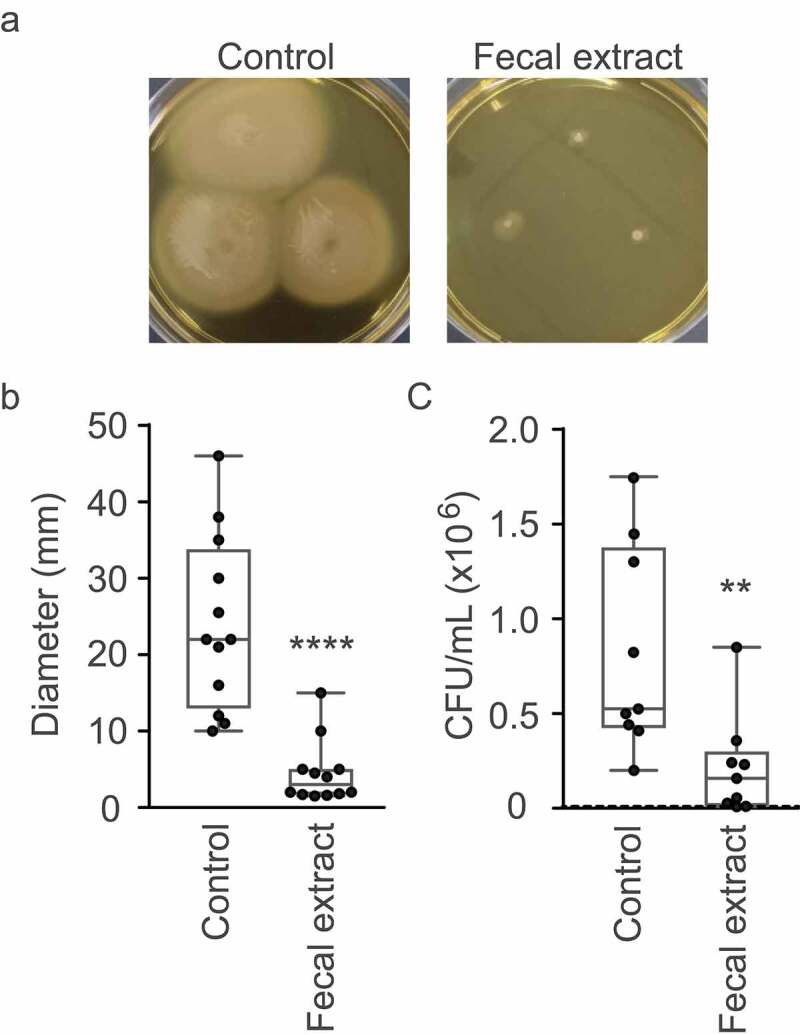
Effect of the fecal extract on *V. cholerae* motility and mucin penetration

The RNAseq results described above suggested that the human gut metabolome contains molecules that may inhibit *V. cholerae* motility. To confirm this hypothesis, we performed phenotypic tests to assess swimming motility of *V. cholerae* when grown in the absence or presence of the fecal extract in BHI medium with 0.3% agar. As can be observed in Figure 5a and Figure 5b, the fecal extract severely inhibited *V. cholerae* motility, with a reduction of 5.3-fold in the diameter of motility halos in the presence of the fecal extract when compared to the control. This confirms that the modulation of motility and chemotaxis gene expression elicited by the fecal extract translated into a significantly reduced swimming capacity. We also investigated the effect of the fecal extract on mucin layer penetration *in vitro*. Motility is an important trait for enteric pathogens, as they must navigate the intestinal lumen to reach their preferred colonization niche. In the case of *V. cholerae*, it must traverse the thick mucus layer that covers the intestinal epithelium to reach and attach to host cells. Therefore, we measured the ability of *V. cholerae* cells to traverse a mucin column when exposed to the fecal extract. The number of bacteria recovered from the bottom of columns after 30 min of incubation was 15 times lower when *V. cholerae* was grown in the presence of the fecal extract (Figure 5c), thereby showing that bioactive molecules from the fecal metabolome significantly hamper the ability of *V. cholerae* to penetrate mucin.

A. BHI agar plates (0.3%) with or without the addition of the fecal extract were inoculated with *V. cholerae*, incubated for 16 h, and then photographed. B. Motility zone measurements (in mm). C. *V. cholerae* cells (1.5x10^7^ CFU) grown in the absence or presence of the fecal extract were loaded on top of 1% mucin columns and were allowed to penetrate for 30 min. Bacteria were collected from the bottom half of the columns, serially diluted and plated on BHI agar for enumeration. Results shown represent means and standard errors of eight biological replicates (n = 8). The dashed line in C represents the limit of detection. **p< .01; ****p< .001.

### The human gut metabolome promotes V. cholerae biofilm formation

**Figure 6.**
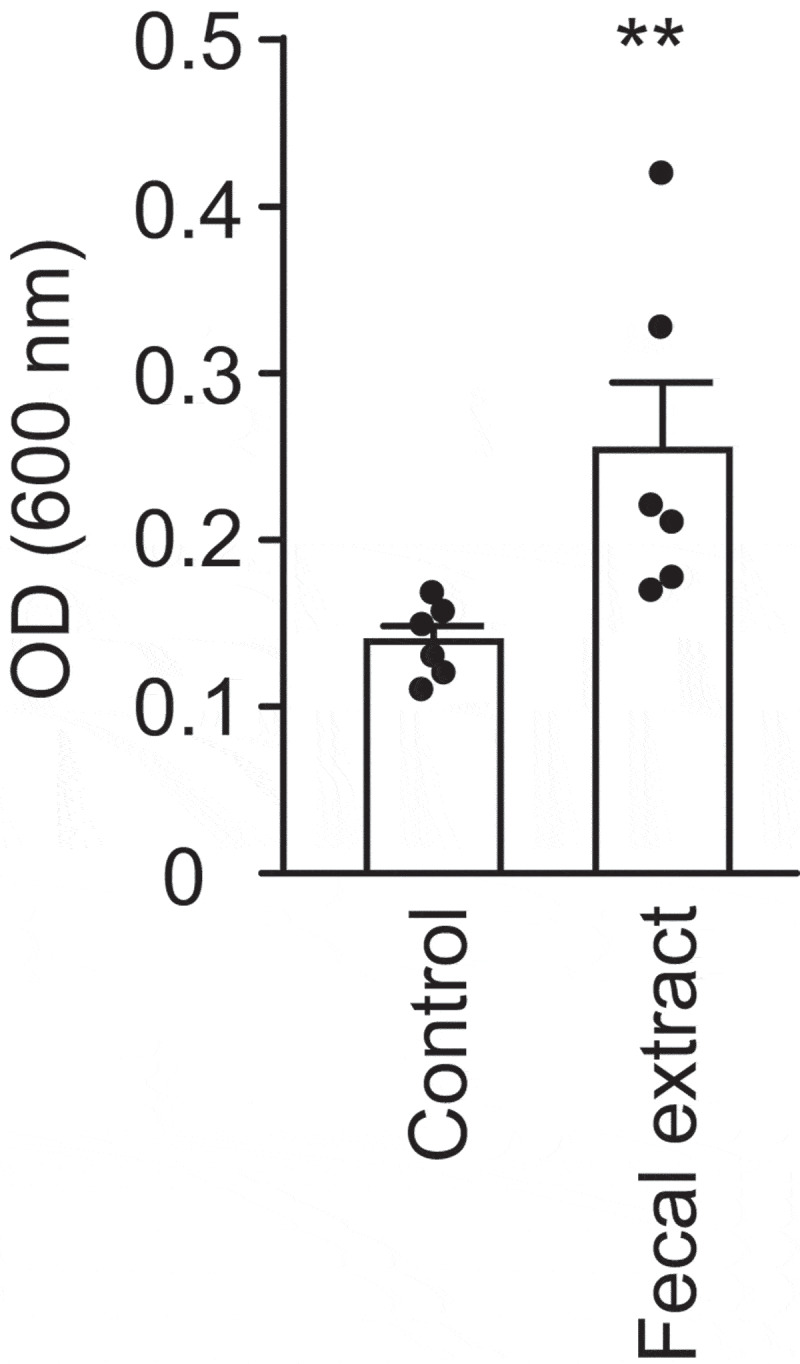
Effect of a fecal extract on *V. cholerae* biofilm formation

Planktonic and sessile bacterial behaviors are commonly subjected to shared regulatory control, where the expression of genetic requirements for one of these behaviors is usually associated with the repression of the other. Therefore, since the fecal extract strongly repressed *V. cholerae* motility, we investigated if the extract could affect *V. cholerae* biofilm formation. As shown in Figure 6, *V. cholerae* growth in the presence of a fecal extract increased surface association and biofilm formation by 1.8-fold. However, total bacterial biomass (biofilm + planktonic) was slightly reduced by growth in the presence of the fecal extract (control: 0.843 ± 0.078; extract: 0.737 ± 0.089), indicating that the increase in biofilm formation observed was not due to an increase in growth.

*V. cholerae* was grown in LB broth in the absence or presence of a fecal extract. After 48 h of incubation at 37 °C, biofilm cells were harvested and quantified. **p< .01.

### Gut microbiota members produce bioactive molecules that modulate V. cholerae motility and mucin penetration

**Figure 7.**
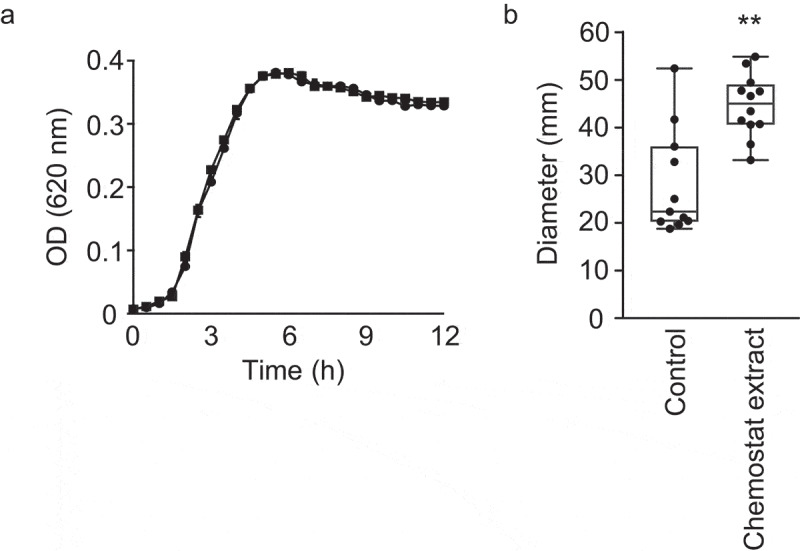
Impact of chemostat extract on *V. cholerae* growth and motility *in vitro.*

After determining that the human gut metabolome contains compounds that inhibit *V. cholerae* motility, we sought to establish whether the microbiota, and not the host, was responsible for this activity. Also, we sought to determine which component of the gut microbiota could be the source of the bioactive compound. For that purpose, we cultured a highly complex, defined microbial community containing 124 bacterial strains comprising 69 distinct species (Table S2) in a chemostat reactor designed to mimic the conditions encountered by the human gut microbiome.^33^ After community stabilization, we extracted small molecules from this consortium and tested the effect of this extract on *V. cholerae* growth and swimming motility. *V. cholerae* growth was completely unaltered in the presence of this extract (Figure 7a). Interestingly, however, we found that the community extract caused a modest but statistically significant increase (1.6-fold) in *V. cholerae* swimming motility (Figure 7b).

A. Cultures were grown in BHI broth with or without an extract from chemostat cultures at 37 °C and growth was measured by absorbance (OD_620_) for 12 h in a microplate reader. Results represent the average of three independent measurements (n = 3), and bars show the standard errors of means. Circles indicate control cultures, whereas squares represent cultures containing the chemostat extract. B. BHI agar plates (0.3%) with or without the addition of the chemostat extract were inoculated with *V. cholerae* and incubated for 16 h. Motility zones were then measured (in mm). Results represent the average of twelve independent measurements (n = 12) and bars show the standard errors of means. **p< .01.

**Figure 8.**
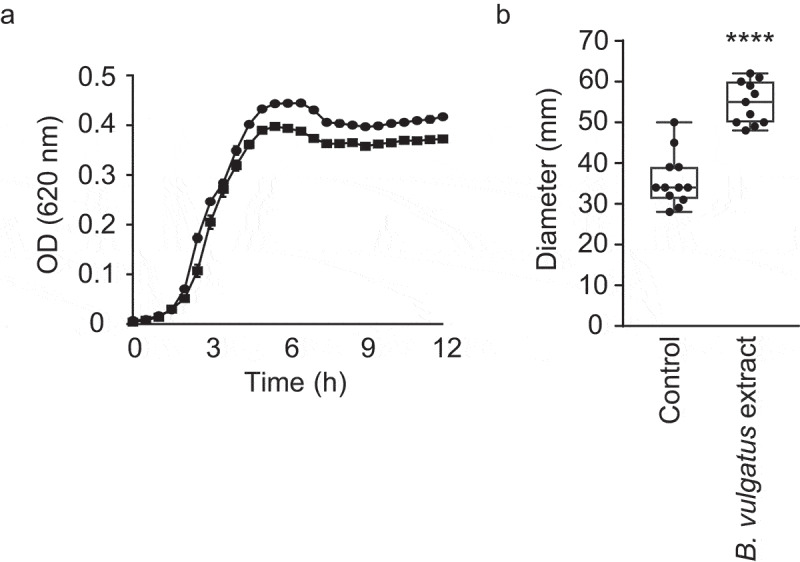
Impact of small molecules produced by *B. vulgatus* on *V. cholerae* motility and growth

Given that a small-molecule extract from a complex microbial community induced *V. cholerae* motility, we sought to identify members of this community that could be involved in the induction of motility. Recently, You *et al*. showed that *B. vulgatus* has a critical role in protecting adult mice from *V. cholerae* infection, and that several microbiota-derived small molecules with growth-inhibitory activity against *V. cholerae* may be involved, supporting the notion that chemical interplay between the microbiota and *V. cholerae* is commonplace.^34^ Although it is not the same strain as the one used by You and coworkers, the chemostat community described herein did contain a *B. vulgatus* strain. Therefore, we sought to determine if *B. vulgatus* produces small extracellular compounds that affect *V. cholerae* motility. Indeed, growth in the presence of a *B. vulgatus* extract caused a significant, 1.5-fold increase in *V. cholerae* motility, reminiscent of the increase caused by the extract from the complex community grown in the laboratory (1.6-fold; Figure 8a). Again, *V. cholerae* growth was completely unaltered in the presence of this extract (Figure 8b).

A. Cultures were grown in BHI broth with or without the addition of a *B. vulgatus* extract at 37 °C and growth was measured by absorbance (OD_620_) for 12 h in a microplate reader. Results represent the average of three independent measurements (n = 3), and bars show the standard errors of means. B. BHI agar plates (0.3%) with or without the addition of the *B. vulgatus* extract were inoculated with *V. cholerae* and incubated for 16 h. Motility zones were then measured (in mm). Bars show the standard errors of means. ****p< .001.

**Figure 9.**
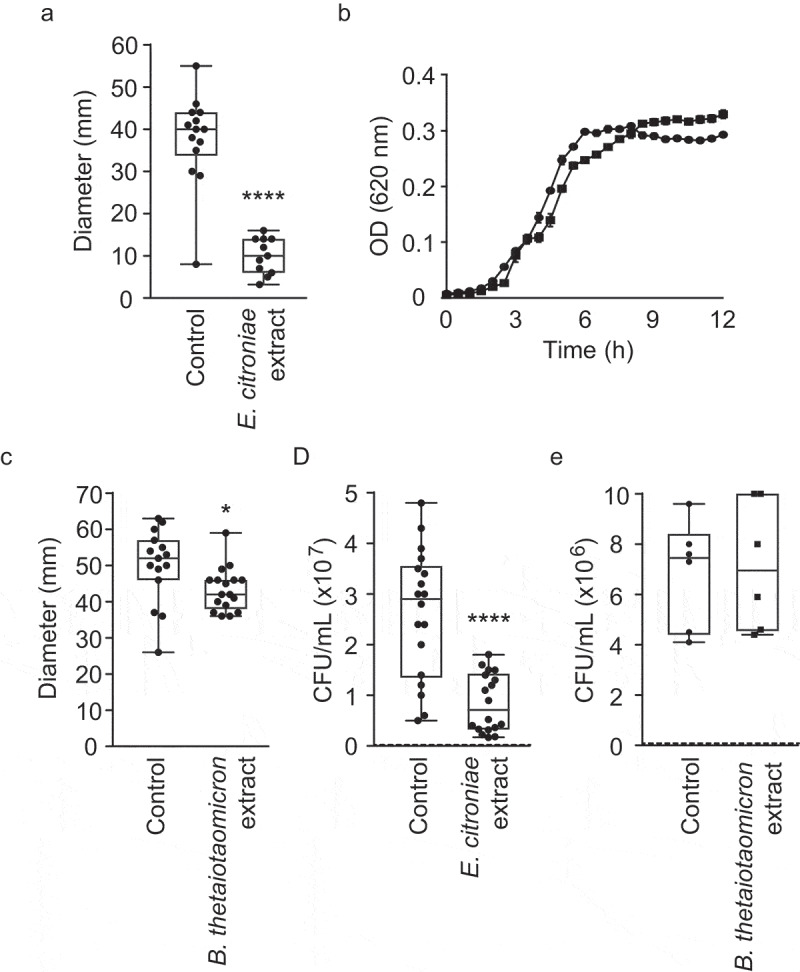
Impact of small molecules produced by *E. citroniae* and *B. thetaiotaomicron* on *V. cholerae* growth, swimming motility, and mucin penetration

Although the presence of *B. vulgatus* in the defined community may explain its motility-enhancing effect, the negative impact of the human fecal extract on *V. cholerae* motility remained to be understood. Previously, we identified a specific member of the human gut microbiota, *E. citroniae*, that produces a bioactive compound when grown in pure culture that inhibits *S. enterica* host cell invasion gene expression.^11^ Therefore, to test if *E. citroniae* could recapitulate the repression of *V. cholerae* motility observed with the fecal extract, we cultured one of the bioactive *E. citroniae* strains previously described (FM-V5-E) and tested the effect of secreted compounds on *V. cholerae* motility and growth. As can be seen in Figure 9, *E. citroniae* metabolites led to potent inhibition (3.8-fold) of *V. cholerae* motility (Figure 9a) but showed no significant effect on *V. cholerae* growth (Figure 9b). As a comparison, we tested extracts from similarly grown cultures of *B. thetaiotaomicron* (ATCC29741). This species was chosen because it is a highly-abundant, highly-prevalent member of the human gut microbiota.^35–37^ Additionally, this species has been shown to produce small molecules with impact on enteric pathogen virulence gene expression.^13,38^ Figure 9c shows that, although statistically significant, the effect of a *B. thetaiotaomicron* extract on *V. cholerae* motility was negligible (1.2-fold). This phenotype was further confirmed by mucin penetration assays using both *B. thetaiotaomicron* and *E. citroniae* extracts. While *B. thetaiotaomicron* had virtually no impact on mucin penetration by *V. cholerae*, an *E. citroniae* extract inhibited mucin penetration 3.1-fold (Figure 9d and Figure 9e).

BHI agar plates (0.3%) with or without the addition of *E. citroniae* (A) or *B. thetaiotaomicron* (C) extracts were inoculated with *V. cholerae* and incubated for 16 h. Motility zones were then measured (in mm). Bars show the standard errors of means. *p< .05; ****p< .001. B. Cultures were grown in BHI broth with or without *E. citroniae* extracts and growth was measured by absorbance (OD_620_) for 12 h. Results represent the average of three independent measurements (n = 3), and bars show the standard errors of means. D and E. *V. cholerae* cells (1.5x10^7^) grown in the presence of *E. citroniae* (D) or *B. thetaiotaomicron* (E) extracts were loaded on top of 1 mL mucin (1%) columns and were allowed to penetrate. Bacteria were collected from the bottom of the columns, serially diluted and plated on BHI agar for enumeration. Results shown represent the means and standard errors of six biological replicates (n = 6). The dashed lines in D and E represent the limit of detection. *p< .05.

**Figure 10.**
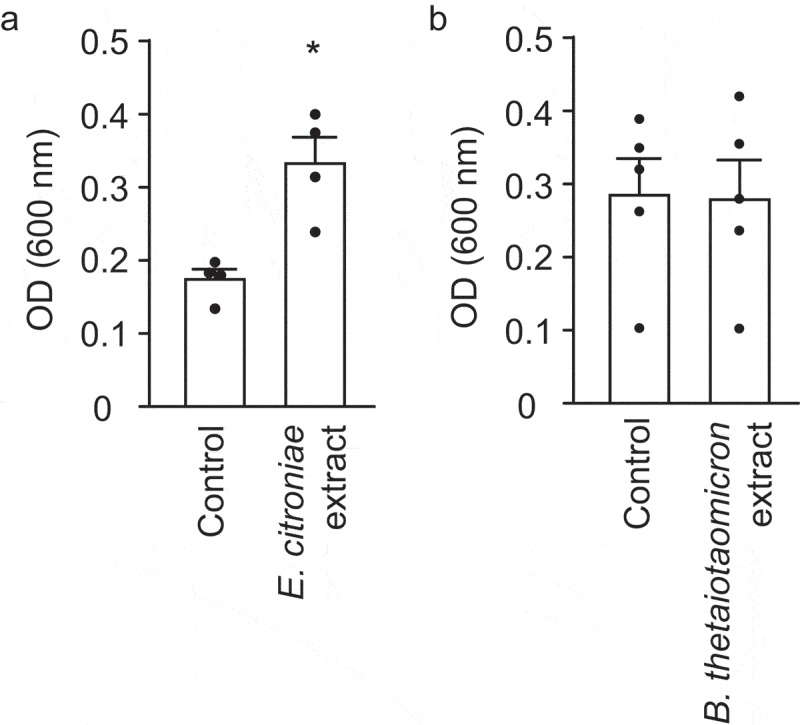
Effect of *E. citroniae* and *B. thetaiotaomicron* extracts on *V. cholerae* biofilm formation

Biofilm formation analyses showed that *B. thetaiotaomicron* extracts do not alter the ability of *V. cholerae* to form biofilms on surfaces (Figure 10B), although *E. citroniae* extracts significantly (*p*< .05) increased biofilm formation by 1.9-fold (Figure 10A). However, total bacterial biomass (biofilm + planktonic) was unaltered by either extract (*E. citroniae* control: 0.751 ± 0.108; *E. citroniae* extract: 0.74 ± 0.043; *B. thetaiotaomicron* control: 0.763 ± 0.085; *B. thetaiotaomicron* extract: 0.753 ± 0.045;), indicating that the increase in biofilm formation was not due to an effect on growth.

*V. cholerae* was grown in LB broth in the absence or presence of *E. citroniae* (A) and *B. thetaiotaomicron* (B) extracts. After 48 h of incubation at 37 °C, biofilm cells were harvested and quantified. *p< .05.

### Statistical analyses

The results are reported as means with their respective standard errors. The results were analyzed using two-tailed Student´s *t*-test in GraphPad Prism 6. Differences were considered significant if the *p* value was less than 0.05.

## Discussion

Bacteria rely heavily on small molecules to interact with their environment. Such compounds function as cues to inform cells of the conditions encountered in their surroundings. Also, they can perform dedicated signaling functions, and the production of and response to these signals – small compounds of diverse chemical properties – is used by most bacteria to regulate their behavior and that of neighboring cells.^39,40^ Interestingly, these compounds are also used for interkingdom communication, and examples of microbe-host interactions that occur through small molecules and signaling mechanisms continue to be reported.^41–44^ Recently, Uchimura *et al*. demonstrated that there is an extensive penetration of a broad range of bacterial small molecules into host tissues, suggesting that the effects of microbial compounds on host physiology are likely to be much larger than currently appreciated.^7^

Using metabolomics, we have previously shown that the human gut is teeming with small molecules, and that many of the molecules detected may represent new compounds.^45^ Some of the metabolites produced by members of the gut microbiota have biological activity, exerting strong effects on pathogen virulence gene expression. Previously, we reported that small molecules present in organic extracts of human feces display antivirulence activity against *S. enterica*.^11^ Interestingly, metabolites produced by strains of *E. citroniae*, a member of the gut microbiota, caused potent inhibition of invasion gene expression by this enteric pathogen. Other studies have shown that *B. thetaiotaomicron*, a predominant member of the human gut microbiota, can inhibit Shiga toxin production by enterohemorrhagic *E. coli* (EHEC) O157:H7.^13,38^ The *Bacteroides* genus represents one of the most important members of the human gut microbiota, and accounts for 10 to 30% of the total bacterial population.^35^ These and other studies demonstrate that small molecule-dependent interactions between members of the human microbiota and invading pathogens may be prevalent, and that such interactions could be involved in the role played by the gut microbiota in protecting the host against enteric infections.

Here, we hypothesized that small molecules derived from the gut microbiota may have significant effects on *V. cholerae*. We began investigating this by analyzing the effect of small molecules from organic extracts of human feces on *V. cholerae* growth. The microbiota can directly inhibit the growth of diarrheal pathogens through inhibitory structures that depend on cell contact, such as the type VI secretory system or by secreting bacteriocins (antimicrobial peptides) or small molecules, such as secondary bile acids.^46–48^ Our results show that small molecules extracted from feces of a healthy donor have a modest effect on *V. cholerae* growth (maximum of 36.6% growth reduction), suggesting that molecules with biological activity against this pathogen are present in the fecal metabolome, although the mechanism for this effect on growth is still unknown. This effect is somewhat similar to what was observed with *S. enterica*.^11^ Interestingly, Silva *et al*. have previously studied the effect of fecal samples as well as bacterial strains cultured from a fecal sample on *V. cholerae* growth, and identified *Peptostreptococcus* sp. and *Lactobacillus* sp. that secreted unidentified metabolites that inhibited the growth of *V. cholerae in vitro* and *in vivo*.^49^ Therefore, it is possible that the mild effect observed in our growth curves could be due to a microbiota-derived inhibitory compound, although further studies are required to support this hypothesis.

Transcriptome analyses revealed a pleiotropic effect of small molecules from human feces on *V. cholerae* gene expression, with almost 25% of the genome being differentially expressed in the presence of the fecal extract. Although the fecal metabolome is rich in chemical diversity, the magnitude of this effect on *V. cholerae* was unexpected, given that our similar study using *S. enterica* revealed differential expression of approximately 5% of the genome.^11^ RNAseq results demonstrated an activation of the sulfate assimilation pathway in the presence of the fecal extract. Small-scale reduction of sulfate by this route is used by several organisms, not just those classified as sulfate reducers, being important for the synthesis of sulfur-containing biomolecules.^50^ In a study by Sigalevich *et al*., it was observed that the transition from anaerobic to aerobic conditions affects the rate of sulfate reduction in *Desulfovibrio oxyclinae*.^51^ Although *V. cholerae* is not a sulfate-reducing microorganism, we observed a significant increase in the expression of genes related to the assimilation of sulfate in the presence of the fecal extract. As this occurred during growth in an atmosphere rich in oxygen and under agitation, we hypothesize that some metabolites present in the extract may induce the activation of a pathway normally associated with an anaerobic environment, as the one seen in the gut.

As we have previously shown, some microbiota species produce molecules that can dampen virulence attributes of enteric pathogens. When *S. enterica* is grown in the presence of small molecules extracted from human feces, the expression of genes coding for one of its type-3 secretion systems, which are essential for its ability to invade host cells and colonize the intestine, is reduced. The ability to invade human epithelial cells *in vitro* is also decreased in the presence of a human fecal extract.^11^ A key component of the early phase of intestinal colonization by *V. cholerae* is motility, as well as the associated chemotaxis.^52^ In this work, we show that 44% of genes involved in *V. cholerae* motility were downregulated in the presence of the fecal extract. Understanding the precise role of chemotaxis in *V. cholerae* colonization is complicated by the presence of three chemotaxis operons (cluster I, II and III) and at least 45 methyl-accepting chemotaxis proteins, MCPs.^53^ Only one of the chemotaxis operons (cluster II) is required for standard chemotaxis.^54^ The function of the other two chemotaxis operons is not presently known. In this work, genes belonging to the three clusters were affected by the fecal extract, with inhibition, although weak, of cluster II gene expression. Many intestinal pathogens rely on chemotaxis, amongst many other factors, to aid in their virulence strategies, and chemotaxis may increase pathogen fitness and virulence in the gut.^55^ Phenotypic analysis to verify if the downregulation of *V. cholerae* motility genes observed affected its swimming ability showed that the fecal extract drastically reduces motility *in vitro*.

In a previous study, Klose and Mekalanos (1998) showed that although *V. cholerae* has five flagellins – FlaA, FlaB, FlaC, FlaD, and FlaE – only FlaA is essential for motility; a strain containing a mutation in *flaA* does not produce a flagellum and is therefore nonmotile.^56^ By RNAseq analysis we observed that *flaA* was repressed in the presence of the fecal extract, which may be one of the reasons for the reduction of *V. cholerae* motility in the presence of this extract. In addition, other flagellar genes important for bacterial locomotion were also downregulated, such as the motor genes *motX, motA*, and *motB.*

The impact of small molecules produced by specific components of the gut microbiota on *V. cholerae* motility was also analyzed. Our results showed that small molecules from different bacterial species have different effects on *V. cholerae* motility. While *B. thetaiotaomicron* extracts did not elicit significant effects on *V. cholerae, B. vulgatus* extracts significantly increased motility. You *et al*. recently described a *B. vulgatus* strain from the murine microbiota that displayed a protective effect on host susceptibility against *V. cholerae* infection.^34^ As the authors showed, *B. vulgatus* suppressed *V. cholerae* colonization of the adult mouse intestine, and they attributed this effect to the production of inhibitory molecules. In our work, we did not observe an effect of *B. vulgatus* on *V. cholerae* growth. However, swimming motility was increased in the presence of the extract, a phenotype that was also observed when *V. cholerae* was cultured in the presence of an extract from a complex microbial community cultured *in vitro*.

The induction of *V. cholerae* motility by extracts from the 124-strain defined chemostat community was unexpected. We have previously shown that *in vitro* complex communities are able to recapitulate the impact of the human fecal metabolome on *S. enterica* virulence gene expression.^11^ Therefore, we set out to reconstitute a complex and diverse *in vitro* community to test its effect on *V. cholerae* motility, expecting that a phenotype similar to the one obtained with fecal extracts would be observed. Aside from the fact that *S. enterica* and *V. cholerae* will likely have different regulatory mechanisms involved in the response to microbiome-derived small molecules, one of the differences between the two experiments is that in the case of *S. enterica* chemostats were inoculated with human feces, whereas isolated strains were used to assemble the defined community described herein. Therefore, it is likely that the former communities were a better representation of those found in the human gut. In that sense, it is possible that the community described herein lacked strains with the ability to repress *V. cholerae* motility. Although the defined community tested did contain an *E. citroniae* strain, this is not the same strain previously shown to have activity against *S. enterica* and shown here to inhibit *V. cholerae* motility (FM-V5-E). We chose not to use FM-V5-E in the chemostat community because during the establishment of complex microbial communities different strains co-adapt to maintain community structure and function. Therefore, we assembled a community of species and strains isolated from the same donor. As previously shown, communities assembled using strains from different donors can lose some of its members and generate a metabolic output that is significantly different from communities assembled using strains from the same donor.^57^ Therefore, inserting FM-V5-E in a community composed of strains from a different donor would have unpredictable effects.

Another potential reason for the discrepancy between results obtained with fecal versus chemostat extracts is that the chemostat community may contain strains (*B. vulgatus* strains and others) whose motility-inducing effects counteract the inhibitory effect observed with *E. citroniae*. Also, although complex and diverse, it is important to stress that the microbial community grown in the chemostat was composed of 69 different species selected from a collection of strains previously isolated from a healthy donor. Therefore, they do not fully represent the microbial diversity found in fecal samples. Further supporting this notion, we showed that, although fecal extracts moderately inhibited *V. cholerae* growth, chemostat extracts have virtually no effect on growth. Therefore, it is clear that the microbial and chemical composition of these two matrices (fecal *vs*. chemostat extracts) is significantly different. The difference observed in their ability to inhibit *V. cholerae* growth likely comes from the fact that fecal extracts will inevitably display higher chemical complexity. For instance, chemostat extracts probably lack various host factors that are likely present in fecal extracts, such as antimicrobial peptides. Thus, the absence of certain strains and compounds in the chemostat community can have unpredictable effects on the net biological activity of the chemostat extracts. Altogether, these data suggest that the effect of the fecal extract on *V. cholerae* motility observed is likely to represent the combined effect of multiple bioactive compounds whose ‘messages’ are somehow integrated into *V. cholerae* regulatory circuits. Nevertheless, we were able to detect a bacterial species that, when grown in pure culture, could recapitulate the effect of the fecal extract. Although *E. citroniae* is a minor member of the human gut microbiota, it has strong antivirulence activity against *S. enterica*. Whether the same molecule exerts the observed effects on *S. enterica* and *V. cholerae* remains to be determined. It is also important to point out that *E. citroniae* may not be the exact bacterial species responsible for the bioactivity of the fecal extract. It is possible, and very likely, that other strains present in the human gut microbiota will have motility-inhibiting activities, although this remains to be determined. Nevertheless, our results using *E. citroniae* establish that members of the microbiota can inhibit *V. cholerae* motility independently of host factors and will facilitate the purification and identification of the bioactive compound.

Bacterial chemotaxis and motility are often required for bacterial pathogenesis, as these processes allow cells to reach their niche, detect nutrients and sense cues from other bacteria or the host.^58^ Thus, bioactive molecules that can inhibit these processes may hold therapeutic promise, since they may prevent the establishment of infections. Studies with *V. cholerae* have shown that, to cause infection, cells need to swim through the intestinal lumen, traverse the mucus layer, and then colonize epithelial cells.^32^ The ability of *V. cholerae* to penetrate mucin is impaired in the presence of fecal and *E. citroniae* extracts, suggesting that small molecules present in these extracts may affect host colonization. Hindrance of *V. cholerae* virulence by these small molecules may allow the use of such molecules in the development of therapeutics. As an alternative, strains that produce the bioactive compound could be used as probiotics to lower burden of *V. cholerae* infection and therefore prevent or treat cholera. Such strains could be either gut microbiota isolates from healthy subjects or engineered strains with desirable properties. Interestingly, antivirulence activity of the probiotic fermented beverage Kombucha against *V. cholerae* has been shown. In this case, small molecules present in the beverage were shown to inhibit motility, alter motility gene expression and inhibit mucin penetration *in vitro*.^59^ The idea of developing new therapeutic alternatives for cholera based on the gut microbiota is particularly enticing in the context of this work, since the *V. cholerae* strain used herein is resistant to multiple antibiotics, and a non-antibiotic therapeutic intervention would be widely useful during infections with drug-resistant organisms. However, further studies will be required to address the therapeutic value of such compounds, and to reveal the details of interspecies interactions in the human microbiota and how they contribute to the balance between host colonization resistance and susceptibility.

## Supplementary Material

Supplemental Material
