# Supplementary material for: Bioactive small molecules produced by the human gut microbiome modulate Vibrio cholerae sessile and planktonic lifestyles
Source: Gut Microbes. 2021 May 19;13(1):1918993. doi: 10.1080/19490976.2021.1918993 (PMC8143261; doi:10.1080/19490976.2021.1918993)
Supplement: Supplemental Material [file KGMI_A_1918993_SM4398.zip › Supplementary information/Figure S1.pdf]

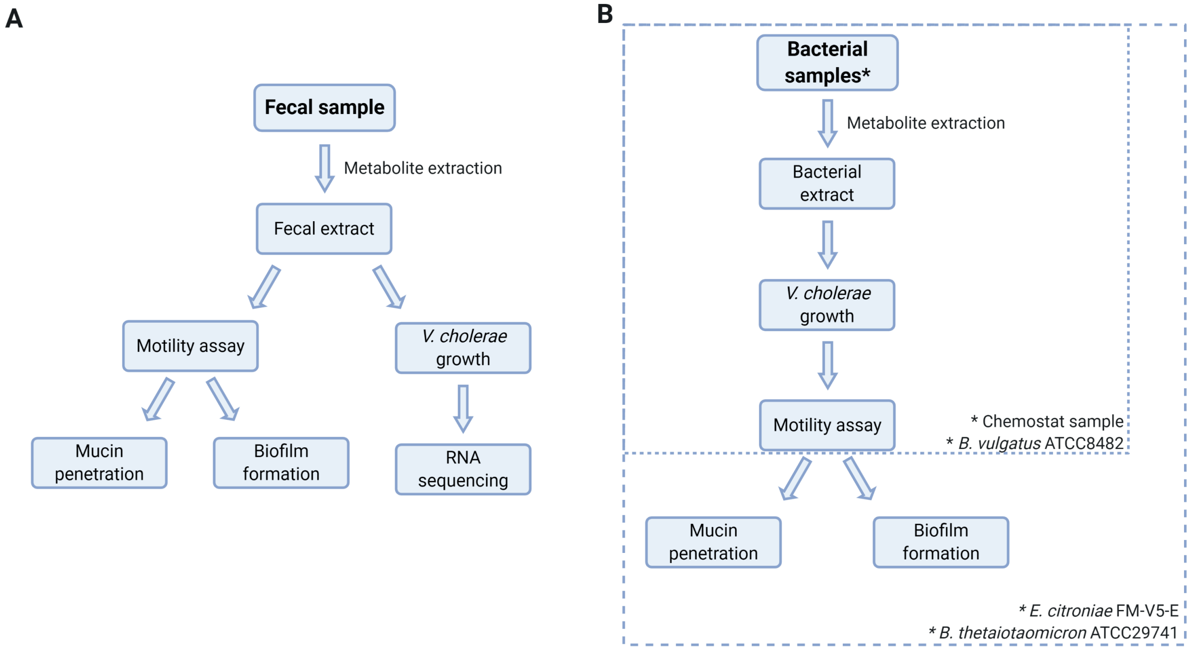

Figure S1. Schematic of the methods employed in this work. A. experiments using fecal extracts. B. Experiments using laboratory cultures.
